# Genetic Testing in Various Neurodevelopmental Disorders Which Manifest as Cerebral Palsy: A Case Study From Iran

**DOI:** 10.3389/fped.2021.734946

**Published:** 2021-09-03

**Authors:** Marzieh Nejabat, Soroor Inaloo, Afsaneh Taghipour Sheshdeh, Shima Bahramjahan, Fatima Masoomi Sarvestani, Pegah Katibeh, Hamid Nemati, Seyed Mohammad Bagher Tabei, Mohammad Ali Faghihi

**Affiliations:** ^1^Pediatric Neurology Ward, Shiraz University of Medical Sciences, Shiraz, Iran; ^2^Neonatal Research Center, Shiraz University of Medical Sciences, Shiraz, Iran; ^3^Persian BayanGene Research and Training Center, Shiraz University of Medical Sciences, Shiraz, Iran; ^4^Epilepsy Research Center, Shiraz University of Medical Sciences, Shiraz, Iran; ^5^Shiraz Neuroscience Research Center, Shiraz University of Medical Sciences, Shiraz, Iran; ^6^Maternal-Fetal Medicine Research Center, Shiraz University of Medical Sciences, Shiraz, Iran; ^7^Express Gene Molecular Diagnostics Laboratory, Palmetto Bay, FL, United States

**Keywords:** atypical cerebral palsy, next-generation sequencing, motor disabilities, neuroimaging, developing brain

## Abstract

**Purpose:** Cerebral palsy (CP) is a heterogeneous permanent disorder impacting movement and posture. Investigations aimed at diagnosing this disorder are expensive and time-consuming and can eventually inconclusive. This study aimed to determine the diagnostic yield of next generation sequencing in patients with atypical CP (ACP).

**Methods:** Patient eligibility criteria included impaired motor function with onset at birth or within the first year of life, and one or more of the following conditions: severe intellectual disability, positive family history, brain imaging findings not typical for cerebral palsy, abnormal neurometabolic profile, intractable seizure, normal neuroimaging despite severe psychomotor disability, after pediatric neurologist assessment including neuroimaging and biochemical-metabolic study offered for genetic study.

**Results:** Exome sequencing was done for 66 patients which revealed pathogenic, likely pathogenic, and variants of unknown significance in 36.2, 9, and 43.9%, respectively. We also found 10 new mutations and were able to suggest specific and personalized treatments for nine patients. We also found three different mutations with different phenotypical spectrum in one gene that have not been reported for cerebral palsy.

**Conclusion:** An accurate history and physical examination and determination of patients with atypical cerebral palsy for doing exome sequencing result in improved genetic counseling and personalized management.

## Introduction

Cerebral palsy is a group of chronic neurodevelopmental disorders that is the most common cause of childhood physical disability and shows heterogeneity in all of its aspects including etiology, presentation, functional severity, comorbidities, treatment options, and outcomes (1–4). Cerebral palsy definition derived from Swaiman's pediatric Neurology 2017 (6th Edition). Few cases are solely due to prematurity or severe hypoxic-Ischemia at birth (5, 6). Cerebral palsy is a non-progressive, but often changing, motor impairment syndrome secondary to brain lesions or anomalies in the early stages of its development (7). CP rates have remained the same for 50 years despite major advances in the obstetrics and neonatology. It is seen in around 2–2.5 for every 1,000 births. Although there have been small statistical fluctuations in the cerebral palsy rates amongst children born preterm, the rates of cerebral palsy at term remain stable (5). Along with motor disabilities, children with CP have disturbances of sensation, perception, learning, and behavior. CP imposes great demands on health, social, and educational services, as well as a large financial and emotional burden on families (1–4, 6). In this report we present our experience with a group of patients who were assessed at our institution with neurodevelopmental disorders and initial diagnosis of CP, but in whom the condition was not associated with known perinatal complications or with the brain lesions commonly related to CP. Atypical CP included: full term neonate without history of perinatal and postnatal insult; absence of brain MRI finding compatible with neonatal asphyxia; progressive neurological deterioration; severe or profound intellectual disability; severe hypotonia opposite spasticity; positive family history of one or more similarly affected relatives (8). The main goal of this work was to delineate the clinical manifestations, laboratory data and molecular findings of patients who are regarded as CP mimics or atypical CP, so that more targeted approaches to the diagnosis and management of this condition can be developed, and genetic counseling can further be provided to the families.

## Materials and Methods

The study was approved by the Namazi Hospital, Shiraz University of Medical Science Ethics board. Each patient guardian provided informed consent for study participation and subsequent publication of established results. Exome sequencing was done for 66 patients and WES were not obtained for their parents.

Indeed these patients suffer various neurodevelopmental disorders, some of them presented with atypical cerebral palsy phenotype. The most important complaint of our patients and the reason for their referral was physical disability and delayed motor development and in most of them it has been accompanied by significant degrees of cognitive disability.

This represents a descriptive-analytical cross-sectional retrospective study of patients diagnosed with CP without history of perinatal injury and asphyxia (especially result NICU or prolonged neonatal ward admission by delivery chart review), brain MRI compatible with HIE (7), assessed by pediatric neurologist in the pediatrics clinics of the Shiraz University of Medical Science from 2016 to 2020 years.

The population study is children and adolescents (ages 6 months to 18 years) with delayed motor development from the birth or early infancy assessed by a pediatrician and then referred to a pediatric neurologist.

History and physical examination, brain MRI imaging and laboratory tests were recommended, which included metabolic tests (serum amino acids, urine organic acids, urine and serum acyl carnitines) and in certain cases, such as autism, other metabolic tests such as creatine and purine pyrimidine panel. The patients had clinical and brain imaging (Red flags) findings that led us to perform genetic testing.

For the studied patients, 5 cc of peripheral blood was collected in EDTA tube. After DNA extraction, whole exome sequencing was conducted using Illumina HiSeq 4,000 sequencing platform. Various bioinformatics tools and databases such as ANNOVAR, GATK, and BWA aligner were used for the bioinformatics analysis of the WES results.

Pathogenic and likely pathogenic variants were defined according to the standards and guidelines recommended by the American College of Medical Genetics and Genomics and the Association for Molecular Pathology for the interpretation of genetic variants (9).

### Inclusion Criteria

Non progressive disorder of the development of movement and posture leading to the limitation of activity with onset at birth or within the first year of life.

1. Normal MRI findings despite motor disabilities, atypical white matter lesions or other structural findings that are not typical of CP.
2. Severe symptoms in the absence of a history of perinatal injury.
3. A pattern of disease inheritance, or consanguinity.
4. Isolated muscular hypotonia.
5. Rigidity (as opposed to spasticity) on physician examination.

### Exclusion Criteria

1. Gestational age of 36-week gestation or less.
2. Perinatal complications: asphyxia, respiratory distress syndrome requiring mechanical ventilation, meningitis/encephalitis, non-physiological jaundice.
3. Presence of acquired and/or progressive lesions on brain MRI, such as ischemic lesions, hemorrhage, calcification.
4. Patient with major dysmorphic features and Patients with multiple congenital anomalies.
5. Positive neonatal metabolic screening tests that was confirmed by more accurate tests.

Patients do not have to cover all inclusion criteria at the same time but all of our patients met all the exclusion criteria at the same time.

## Results

A total of 66 affected individual with atypical CP were examined. The general characteristics for all 66 propends are listed in Table 1. Clinical findings, including details of neurologic exam and seizure and intellectual disability and developmental status can be found in Table 2. It should be noted that intellectual disability was the most frequent sign after motor symptoms. Their MRI findings were as follows: normal findings were the most frequent (36.2%), brain atrophy (24.2%), White matter lesion (23%), Neuronal migration defects (7.5%), Vermian and cerebellar hypoplasia (3%), Basal ganglia lesion (1.5%), Corpus callosum agenesis (1.5%), Molar tooth sign (3%).

**Table 1:** Patients general characteristics.

**Characteristics**	**Number (present %)**		
Gender	Female 30 (45.4%)	Male 36 (54.6%)	Total 66 (100%)
Age	6 months to 18 years 49 (74.2%)	Average age 4.02 ± 3.30	
Consanguineous parents	First cousin 49 (74.2%)	Others 9 (13.6%)	Non related 8 (12.1%)
Same affected family member	Siblings 12 (18.1%)	Others 9 (13.6%)	Not affected family member 45 (68.1%)

**Table 2:** Patients clinical finding and developmental status.

**Signs and symptoms**	**Number (%)**
Intellectual disability (ID)	57 (86.3%)
Seizure	32 (48.4%)
Hypotonia	37 (56%)
Hypertonia	25 (37.8%)
Speech defect	27 (40.9%)
Microcephaly	19 (28.7%)
Macrocephaly	5 (7.5%)
Nystagmus	5 (7.5%)
Ataxia	4 (6%)
Autistic behavior	3 (4.5%)
Mild dysmorphy	3 (4.5%)
Global developmental delay	54 (81.8%)
Only motor delay	12 (18.2%)

Different inheritance models of candidate ACP variants have been shown in Table 3. Furthermore, types of pathogenicity of genetic variants in candidate ACP genes have been displayed in Table 4. Missense variants were the most frequent (56%) type. Number of patients with each clinical feature, MRI and metabolic finding evaluated; associated causal genes in Table 5. Detailed results of exome sequencing in our atypical CP patients; associated diseases, causal genes and their location and Inheritance pattern has been described in Table 6.

**Table 3:** Different inheritance models of candidate ACP variants.

**Inheritance model**		**Number of patients (%)**
Autosome Recessive (AR)		50 (75.7%)
Autosome Dominant (AD)		9 (13.6%)
X linked (XL)	XL Dominant (XLD)	2 (3%)
	XL Recessive (XLR)	1 (1.5%)
Exome Negative		4 (6%)

**Table 4:** Types of pathogenicity of genetic variants in candidate ACP genes.

**Variant type**	**Variants number (%)**	**Gene ID**
Variant unknown significant	29 (43.9%)	PIGG, RHOBTB2, SUCLG1, ADGRG1, SPR, TMEM237, ATL1, WWOX(37,24)^*^, LAMA, MECP2, RAPSN,SPEG, FBXL4, LAMB1, SEPSECS, SLC13A5, MTHFR, GABRB1, ST3GAL5, ALS2, PC, GLB1, SCN9A, DPM1, ATP6V1A, DCX, TCAP, SLC6A5
Pathogen	24 (36.3%)	ASPA, TRAPPC4, TRAPPC9, ADGRG1, WWOX42, KIAA0586, MYOT, OCLN, SURF1, GAMT, TDP2, PDHX, LAMA2(60,59)^*^, MOCS1, AP4M1, SCN1A, MYO5A, SUOX, AP3B2, GAN, SNX14, FOXG1
Likely pathogen	6 (9%)	HSD17B4, HEXA, TREX1(31,17)^*^, HACE1, KCNT1
Exome negative	4 (6%)	
Known mutation/Previously report	3 (4.5%)	PIGN, WDR45, PLA2G6

* *patient number*.

**Table 5:** Clinical feature, MRI and metabolic finding evaluated and associated causal genes.

**Clinical feature**	**Genes (and CP family number when more than one family had the same causal gene)**	Number of patients (%)^*^
Abnormal neurological exam (hypotonia, hypertonia, dystonia, ataxia)	PIGN, MTHFR, DPM1, HSD17B4, LAMA259,60, WDR45, DCX, MYOT, HEXA, GLB1, KCNT1, TDP2, WWOX42, GAMT, SURF1, GABRB1, FBXL4, SEPSECS, TMEM237, ASPA, RAPSN, TCAP, AP3B2, SPEG, RHOBTB2, SUOX, PIGG, KIAA0586, S3GAL5, SCN9A, SCN1A, FOXG1 ATP6V1A, MOCS1, ADGRG1, PC, OCLN, SLC6A5, ALDH5A1, WWOX24,37, TRAPPC9, PDHX, PLA2G6, TREX131,17, ALS2, AP4M1, MECP2, ATL1, SPR, ADGRG1, TRAPPC4, SLC13A5, SUCLG1, MYO5A, HACE1, LAMB1, LAMA, SNX14, GAN,	62 (100%)
Intellectual disability	ATP6V1A, PIGN, MOCS1, MTHFR, DPM1, HSD17B4, ADGRG1, LAMB1, PC, WDR45, DCX, OCLN, FOXG1, SCN1A, HEXA, SCN9A, GLB1, KCNT1, TDP2, ALDH5A1, ST3GAL5, GAMT, WWOX24,37,42, TRAPPC9, GABRB1, PDHX, FBXL4, PLA2G6, TREX131,17, SEPSECS, KIAA0586, AP4M1, LAMA, MECP2, TMEM237, SPR, ADGRG1, TRAPPC4, SNX14, SLC13A5, AP3B2, SUCLG1, RHOBTB2, SUOX, PIGG, MYO5A, HACE1, ASPA, MYOT, TCAP	53 (85.4%)
Microcephaly	MOCS1, MTHFR, OCLN, GABRB1, PLA2G6, PDHX, SEPSECS, WWOX24,37,42, MECP2, FBXL4, TRAPPC4, TREX131,17, SUOX, SLC13A5, MYO5A, TRAPPC9	19 (30.6%)
Macrocephaly	PIGN, LAMA260,59, ASPA, HACE1,	5 (8%)
Abnormal biochemical profile	MOCS1, HSD17B4, DCX, GLB1, KCNT1, TDP2, PDHX, FBXL4, ALDH5A1, ASPA, TCAP, SLC13A5, SUOX,	12 (19.3%)
Normal brain MRI with severe or profound ID/neurologic impairment	DPM1, LAMB1, WDR45, SCN1A, MYOT, SCN9A, KCNT1, TDP2, ST3GAL5, GAMT, SURF1, WWOX37, GABRB1, ALS2, KIAA0586, AP4M1, MECP2, ATL1, RAPSN, TCAP, AP3B2, SPEG, SUCLG1, RHOBTB2,	24 (38.7%)
Unusual MRI evidence for CP	ATP6V1A, PIGN, MOCS1, MTHFR, HSD17B4, LAMA260,59, ADGRG1, PC, DCX, OCLN, FOXG1, HEXA, GLB1, PDHX, FBXL4, PLA2G6, TREX131,17, LAMA, TMEM237, ADGRG1, TRAPPC4, ASPA, SNX14, SUOX,	26 (41.9%)
Interactable seizures	ATP6V1A, PIGN, MOCS1, MTHFR, DPM1, PC, DCX, KCNT1, OCLN, WWOX24,37,42, FOXG1, SCN1A, SCN9A, GAMT, ST3GAL5, GALDH5A1, SURF1, PDHX, AP4M1, LAMA, MECP2, ADGRG1 TRAPPC4, ASPA, SLC13A5, AP3B2, RHOBTB2, SUOX, PIGG, MYO5A,	32 (51.6%)
Autistic behavior	SULCG1, LAMA, ATP6V1A	3 (4.8%)
Mild dysmorphy	PIGN, FBXL4, AP3B2,	3 (4.8%)

* *The percentages are based on the total number of patients with clinically relevant genetic findings (62)*.

**Table 6:** Exome sequencing results in selected atypical CP patients.

**Patient No**.	**Gene & transcript**	**Variant**	**Associated disease**	**OMIM**	**Zygosity**	**ACMG**	**CADD**	**Inheritance**
2	HACE1 NM_020771	c.124delC p.Q42Nfs*23	Spastic paraplegia and psychomotor retardation with or without seizures	616756	Hom	Likely pathogen	28.3	AR
3	MYO5A NM_001142495	c.832C>T p.R278X	Griscelli syndrome type1	214450	Hom	pathogen	38	AR
4	PIGG NM_001289052	Exon 5:c.744_747del p.S248fs	Mental retardation, AR 53	616918	Hom	VUS		AR
5	SUOX NM_001032387	c.1585C>T p.R529X	Sulfite oxidase deficiency	272300	Hom	pathogen	33	AR
6	RHOBTB2 NM_001160037	c.1702G>A p.G5685	Epileptic encephalopathy early infantile 64	618004	Het	VUS	27	AD
7	SUCLG1 NM_003849 KIDINS220 NM_020738	c.512A>G p.N171S c.4727C>T p.A1576V	Mitochondrial DNA depletion syndrome 9 Spastic paraplegia, ID, nystagmus, and obesity	245400617296	Hom Het	VUSVUS	28 23	AR AD
8	SPEG NM_005876	c.7598C>T p.S2533L	Centronuclear myopathy 5	615959	Hom	VUS	23.5	AR
9	AP3B2 NM_002491	c.202G>T p.G68X	Epileptic encephalopathy early infantile 48	617276	Hom	pathogen	38	AR
11	SLC13A5 NM_177550	Exon 10:c.1437+1G>T	Early onset Epileptic encephalopathy AR	608305	Hom	VUS	20.7	AR
12	KCNMA1 NM_001271519 TCAP NM_003673	c.2101+1G>A c.113G>T p.C38F	Paroxysmal non-kinesigenic dyskinesia, 3 Muscular dystrophy, limb-girdle, AR	609446601954	Het Hom	PathogenVUS	21 23	AD AR
13	GAN NM_022041	Exon 7:c.1181dupA p.Y394X	Giant axonal neuropathy−1	256850	Hom	pathogen		AR
14	SNX14 NM_153816	Exon 3:c.898G>T p.R111X	Spinocerebellar ataxia AR 20	616354	Hom	pathogen	36	AR
15	RAPSN NM_005055	c.814G>A p.A272T	Myasthenic syndrome congenital, 11	616326	Hom	VUS	32	AR
16	ASPA NM_000049	Exon 1:c.79G>A p.G27R	Canavan disease	271900	Hom	pathogen	33	AR
17,31	TREX1 NM_033629	c.218C>T p.P73L	Aicardi-Goutieres syndrome 1	225750	Hom	Likely pathogen	27.9	AR
18	TRAPPCA4 NM_016146.6	c.454+3A>G	Neurodevelopmental disorder, epilepsy spasticity, Brain atrophy	618741	Hom	pathogen		AR
19	ADGRG1 NM_001145773	Exon11:c1357dup T:p.V452fs	Bilateral frontoparietal Polymicrogyria AR	604110	Hom	VUS		AR
20	SPR NM_003124	Exon 1:G40A p.G14R	Dopa-responsive dystonia due to sepiapterin reductase defiency	612716	Hom	VUS	25.8	AR
21	TMEM237 NM_001044385	Exon 6:c.550dupA p.S176fs	Joubert syndrome 14	614424	Hom	VUS		AR
22	ALT1 NM_015919	Exon 5:c.T526C p.Y176H	Neuopathy hereditary sensory ID, AD; Spasticparaplegia3A, AD (AR?)	606439	Hom	VUS	24	AR
23	MECP2 NM_001110792	Exon3:c.A946G p.K316E	Rett syndrome	300005	Hemizygouss	VUS	20.4	XLD
24	WWOX NM_130791	Exon 3-4 deletion 20kb	Epileptic encephalopathy early infantile 28	605131	Hom	VUS		AR
25	LAMA NM_5559	Exon37: c.5379+1G>T	Poretti-Boltshauser syndrome AR	615960	Hom	VUS		AR
26	AP4M1 NM_004722	c.1225T>C p.F409L	Spastic paraplegia 50 (SPG50)	612936	Hom	NR	26	AR
27	KIAA0586 NM_001244189	Exon5:c.428delG p.R143Kfs*4	Joubert syndrome 23	616490	Hom	Pathogen		AR
28	SEPSECS NM_016955	Exon7:c.G877A p.A293T	Pontocerebellar hypoplasia Type 2D AR	613009	Hom	VUS	33	AR
29	ALS2 NM_020919	Exon8: c.1738-2A>G	Juvenile lateral Sclerosis, Infantile onset ascending spastic paralysis	606352	Hom	VUS		AR
32	PLA2G6 NM_001004426	Exon16:c.T2208G P.y736X,stopgain	Infantile Neuroaxonal dystrophy and brain iron accumulation	603604	Hom	Pathogen(Known)	37	AR
33	PDHX	Exon/Intron boundaryexon8 c.965_ 1023del59bp p.k321fs5*	Pyruvate dehydrogenase complex deficiency Leigh syndrome	312170	Hom	pathogen		AR
27	KIAA0586 NM_001244189	Exon5:c.428delG p.R143Kfs*4	Joubert syndrome 23	616490	Hom	Pathogen		AR
28	SEPSECS NM_016955	Exon7:c.G877A p.A293T	Pontocerebellar hypoplasia Type 2D AR	613009	Hom	VUS	33	AR
29	ALS2 NM_020919	Exon8: c.1738-2A>G	Juvenile lateral Sclerosis, Infantile onset ascending spastic paralysis	606352	Hom	VUS		AR
32	PLA2G6 NM_001004426	Exon16:c.T2208G P.y736X,stopgain	Infantile Neuroaxonal dystrophy and brain iron accumulation	603604	Hom	Pathogen(Known)	37	AR
33	PDHX	Exon/Intron boundaryexon8 c.965_ 1023del59bp p.k321fs5*	Pyruvate dehydrogenase complex deficiency Leigh syndrome	312170	Hom	pathogen		AR
34	FBXL4 NM_012160	Exon8:c.1506_1507insCT p.G503fs	Mitochondrial DNA depletion syndrome 13 (encephalomyopathic type)	615471	Hom	VUS		AR
35	GABRB1 NM_000812	c.1243G>C p.G415R	Epileptic encephalopathy, early infantile,45	617153	Het	VUS	18.9	AR
36	TRAPPC9 NM_031466 SUOX NM_031466	c.2785C>T p.R929X c.739C>A (p.L247M)	Mental retardation autosomal recessive 13 Sulfite oxidase deficiency	613192272300	Hom Hom	PathogenLikely pathogen	41 23.9	AR AR
37	WWOX NM_001291997	c.889G>T p.G297C	Epileptic encephalopathy early infantile 28	616211	Hom	VUS	25.7	AR
38	SURF1 NM_003172	c.845_846del p.S282Cfs*9	Leigh syndrome due to COX IV deficiency	616684	Hom	Pathogen	35	AR
39	GAMT NM_138924	c.491delG p.G164Afs*14	Cerebral creatin deficiency syndrome 2	612736	Hom	Pathogen		AR
40	ST3GAL5 NM_003896	c.584G>A p.C195Y	Salt and pepper developmental regression	609056	Hom	VUS	32	AR
41	ALDH5A1 NM_170740	c.1441+1G>T	Succinic semialdehyde dehydrogenase deficiency	271980	Hom	Pathogen	27	AR
42	WWOX NM_016373	c.220dupT p.V76Cfs*2	Epileptic encephalopathy early infantile 28	616211	Hom	Pathogen		AR
43	TDP2 NM_016614	c.4G>T p.E2X	Spinocerebellar ataxia, autosomal recessive 23	616949	Hom	Pathogen	35	AR
44	KCNT1 NM_020822	c.862G>A p.G288S	Epileptic encephalopathy early infantile 14	614959	Het	Likely pathogen	25	AD
45	GLB1 NM_000404	c.902C>T p.A301V	GM1-gangliosidosis, type 1	230500	Hom	VUS	37	AR
46	SCN9A NM_002977	c.1370G>A p.G457D	Epilepsy, generalized with febrile seizures plus, type7 Dravet syndrome	613863	Het	VUS	23	AD
47	HEXA NM_000520	c.533G>A p.R178H	Tay-Sachs disease	272800	Hom	Likely pathogen	35	AR
48	SLC6A5 NM_001080476	c.922T>C p.W308R	Hyperekplexia 3	614618	Het	VUS	27	AD/AR
49	MYOT NM_006790	c.655C>T p.R219X	Myopathy, spheroid body Myopathy,myofibrillar,3	182920 609200	Het	Pathogen	38	AD
50	SCN1A NM_006920 ERMARD NM_018341	c.1486_1490del p.E496Kfs*20 c.168_169del (p.E57Vfs*19)	Epileptic encephalopathy, early infantile, 6 (Dravet syndrome) Periventricular nodular heterotopia 6	607208 615544	Het Het	Pathogen VUS		AD AD
51	FOXG1 NM_005249	c.563C>A p.A188E	FOXG1 syndrome (Rett syndrome, congenital variant)	613454	Het	Pathogen	33	AD
52	OCLN NM_002535	c.1054C>T p.Q352X	Pseudo-TORCH syndrome 1	251290	Hom	Pathogen	17	AR
53	DCX NM_001195553	Exon3:c.365-1G>A	Lissencephaly and subcortical laminal heterotopia, X-linked	300067	Het	VUS	20.7	XL
54	WDR45 NM_001029896	Exon6:c.397T p.R133X	Neurodegeneration with brain iron accumulation 5 (X-linked Dominant)	300526	Het	Pathogen (previously reported)	18.5	XLD
56	PC NM_000920	c.C2821A p.P941T	Pyruvate carboxylase deficiency	266150	Hom	VUS		AR
57	LAMB1 NM_002291	c.2387C>T p.P796L	Lissencephaly 5	615191	Hom	VUS	26.9	AR
58	ADGRG1	Exon12:c.1426C>T p.R476X	Polymicrogyria, bilateral frontoparietal	606854	Hom	Pathogen	35	AR
59,60	LAMA2	c.4833dupT p.Leu1612SerfsX2	Congenital muscular dystrophy, early onset	607855	Hom	Pathogen		AR
61	HSD17B4	Exon 15 deletion	D-Bifunctioal Protein deficiency	261515	Hom	Pathogen		AR
62	DPM1 NM_003859	c.361C>T p.L121F	Congenital disorder of glycosylation, type Ie	608799	Hom	VUS	26	AR
63	MTHFR NM_001330358	Exon3:c.C523T p.R175C	Homocystinuria due to MTHFR deficiency	236250	Hom	VUS	22.7	AR
64	MOCS 1 NM_005943	c.604_624del p.202_208del	Molybdenum cofactor deficiency A	252150	Hom	Pathogen		AR
65	PIGN NM_012327	Exon11:c.T996G p.1332M	Multiple congenital anomalies-hypotonia-Seizures syndrome 1, Autosomal recessive	614080	Hom	Pathogen	18.6	AR
66	ATP6V1A NM_001690	Exon4:c.A395G p.K132R	Autosomal dominant Infantile Epileptic encephalopathy,	618012	Het	Pathogen	19.32	AD

In this study, 30 patients had proband and parental Sanger confirmation for mutations and 32 patients didn't have. The following genes were confirmed in our patients by Sanger sequencing: *HACE1, SPEG, SLC13A5, TRAPPC4, FBXL4, TDP2, GAMT, LAMB1, OCLN, WWOX (*37*), TREX1 (*31, 17*), SURF1, WDR45, LAMA2 (*59, 60*), MTHFR, MOCS1, DOM1, SEPSECS, GABRB1, KCNT1, AP4M1, PDHX, FOXG1, ATP6V1A, SPR, PIGG, ATL1, LAMA*.

For others families, Sanger confirmation of the identified variant was not carried out but genotype-phenotype correlation was confirmed.

The mutation found in patients 44 and 66 (*ATP6V1A, KCNT1*) was confirmed by Sanger sequencing but was not segregated possibly due to gonadal mosaism. In the study of exome sequencing, 4 patients with cerebral palsy in this investigation (No. 1, 10, 30, 55) were found to have no mutations despite adequate coverage and re-analysis. In other words, they were exome negative. The diagnostic yield for our patients was 93.9%. Four patients (No. 7, 12, 36, and 50) had two pathogenic variants and did not seek further genetic testing of other family members. In four patients, the identified mutation was the same. However, these patients were not related to each other but were of the same ethnic background, *TREX1* (No.31, 17) and *LAMA2* (No.59, 60). In exome sequencing of three patients, pathogenic mutations were found in a same specific gene (*WWOX* gene) but in different locations; *WWOX* was the most common disease-causing gene in this study. The most common genetic causes of atypical cerebral palsy in our study were neurometabolic (16 patients) and epileptic encephalopathies (14 patients). After these two groups of diseases, the most common disorder was neuromuscular diseases with 6 cases (9%) identified to be due to this condition. After that, 3 cases of spastic paraplegia were found in patients (No. 2, 22, and 26) with responsible genes including (*HACE1, ATL1, AP4M1*), respectively, and two cases of cerebellar ataxia in patients (No. 14 and 43), whose responsible genes were *SNX14, TDP2*, respectively. Although in this study we excluded primary microcephaly and dysmorphies and obvious syndromes from the beginning, we found 4 syndromes, three of which were Joubert spectrum syndrome (No. 21, 25, and 27) due to pathogenic variants in *TMEM237, LAMA*, and *KIAA0586*, respectively. Furthermore, one patient with Griscelli syndrome (No. 3) had disease-causing variants in *MYO5A* gene. Novel and Private mutations were found in ten patients (No. 20, 21, 22, 19, 4, 25, 30, 29, 34, and 24).

## Discussion

In our study, the most common clinical sign was intellectual disability with a prevalence of 86.3% and then seizures with a prevalence of 48.4%. Many similar studies examined only the patient's motor symptoms and did not report any degree of intellectual disability or seizures (10, 11). Brain MRI results in our atypical CP patients showed 36% normal findings, 29.2% cerebral atrophy, and 13% white matter lesions in the studied population. The correlation between the results of the metabolic test and the genetic test in our study was 7% (three out of 43 cases), which was lower than the world reports about 20% (10). In Iran, especially in Fars province, Mass Spectrometry (MS/MS) metabolic screening test has been performed since 2018, which screens 44 of the most common causes of metabolic diseases. Individuals confirmed to be affected with these conditions were excluded from this study. Exome sequencing was performed in 66 patients with atypical cerebral palsy. In four patients, the test was negative (exome negative) and positive findings were found in 62 patients and in 62 different genes that indicates significant heterogeneity of the underlying genetic causes of CP. Other studies have confirmed this severe heterogeneity (2, 3, 5, 8, 10).

The most common inheritance pattern was autosomal recessive (75.7%) that was observed in 88% of consanguineous parents in our study. However, in similar studies, autosomal recessive inheritance was 9 and 10% of the patients' parents were relatives (8). These results show the relationship between autosomal recessive inheritance and parental kinship marriage.

The most common type of pathogenic variants found in this study was VUS (43.9%). Although in other studies, only pathogenic or likely pathogenic variants have been reported (12). Most of detected VUS mutations have been confirmed by Sangar sequencing in probands and their parents and good phenotype-genotype correlation has exist and these mutations have had a very low frequency in community, with these three conditions can even be advised to give prenatal diagnosis after careful genetic counseling. The most genetic variant found in patients with cerebral palsy in our study was missense (56%), but in a similar study, a missense mutation (67%) was reported (8). Our patients, despite having VUS and missense mutations, had a good phenotypic-genotypic correlation, and some of them were not referred for further studies (e.g., Sanger confirmation and family segregation and other complementary genetic studies).

In our study, three patients (patients 24, 37, and 42) all had developmental delay, microcephaly, and recurrent seizures, and their parents were first cousin, and all three had a sibling who had died. Patients 24 and 42 had severe motor impairment with a gross motor function classification system (GMFCS) of 5 but patient 37 had a GMFCS of 2. Patients 24 and 37 also had spasticity but patient 42 had hypotonia. Case 24 has a new and private homozygous deletion of exons 3 and 4, which is 20kb long, but the patient's parents did not undergo Sanger sequencing in order to confirm this variant. Patient 37 had a homozygous mutation c.889G> T (p.G297C) which has been confirmed in patients and parents. Patient number 42 has homozygous mutation duplication c.220dupT (p.V76Cfs ^*^ 2) but unfortunately, the patient's parents did not undergo Sanger sequencing for confirmation. Thus, mutations in the *WWOX* gene have led to developmental delay, microcephaly, recurrent seizures, and motor dysfunction, but mutations in different parts of the gene have resulted in varying severity and type of motor dysfunction (spasticity and hypotonia).

We were able to recommend targeted and personalized treatment for 11 patients; KCNT1-related epilepsy (No. 44) quinidine has been used as an off-label anticonvulsant (13, 14), molybdenum cofactor deficiency type A (No. 64) with cyclic pyranopterin monophosphate (cPMP) (15),Succinic semialdehyde dehydrogenase deficiency (No. 41) with vigabatrin (16), cerebral creatin deficiency (No. 39) with creatin monophosphate (17), WWOX gene mutation (N0. 24-42-37) with lithium (18), Dopa-responsive dystonia (No. 20) with levodopa-carbidopa and other dopamine agonists (19), Congenital myasthenic syndromes RAPSYN deficiency (No. 15) with Pyridostigmine and 3,4 DAP (Diaminoprydine) (20), pyruvate dehydrogenase complex deficiency (No. 33) with Ketogenic Diet and Dichloroacetate (21), methylenetetrahydrofolate reductase deficiency (No. 63) with mefolinate (5-Methyltetrahydrofolate) (22).

Overall, Using a strict and accurate criteria for selecting atypical CP patients who are more likely to be genetic, we were able to identify the genetic cause of a significant proportion of the studied patients. This, in turn led to the reduction of psychological stress and guilt of parents. Furthermore, parents with better understanding of the cause of their child's disability are able to make proper decisions for future pregnancies and other family members can also have a better estimate of the risk of this condition in their offspring. Knowing the exact condition their child is affected with, parents can have a better understanding of its prognosis.

Whole exome sequencing (WES) was performed for all 66 patients with atypical cerebral palsy. The diagnostic yield of these genetic investigations was 93.9% for our patients. In various studies published by other researchers, this rate was lower. For example, in the United States, when examining all types of cerebral palsy (typical and atypical), this rate was 32.7% (10), but in another study, the diagnostic efficiency of WES genetic testing in atypical CP patients in the United states was 41% (10) but in another study in US it was 32.7% (10), in Japan this rate is 52.9% (12) and in the Greece 50% (11) and in a joint study of Canada and the United Kingdom, the diagnostic yield of exome sequencing is reported to be 65% (8).

The most important factors in the high diagnostic yield of genetic testing in our research are as follows:

- We set strict Exclusion and Inclusion Criteria to select specific patients with atypical cerebral palsy with a higher probability of being due to a genetic.
- Selection of severe phenotypes, CP patients in our study did not have the usual course of cerebral palsy (which usually improves with rehabilitation and occupational therapy) and often severe and resistant seizures (51.5%) and significant motor and mental disability (83% GMFCS 3 to 5 and 86.3 % had developmental delay), which sometimes showed a progressive pattern in their follow-up.

The financial constraints of patients and the limited assistance of the welfare department made us select CP patients with the highest probability of being inherited or genetic.

The results of our research contribute to the knowledge of the study of the genetics of cerebral palsy. Pathogenic variants located in a specific gene can lead to a wide range of clinical presentations; such as mutations in different locations within the *WWOX* gene, which in three of our patients caused a range of different symptoms of early epileptic encephalopathy type 28. WES is instrumental in enabling the recognition and definition of expanded phenotypes of single-gene disorders. However, it should be noted that it can be challenging to distinguish them from unidentified multi-locus variations. Multi-locus variation-pathogenic variants in two or more disease genes can potentially explain the underlying genetic basis for apparent phenotypic expansion but it is always possible that a pathogenic variant in a yet unknown disease-causing gene may be responsible for a second disease in these cases (8, 23).

Our study had the following limitations: we didn't investigate these patients with other genetic tools such as Array CGH which evaluate deletion-duplications. Furthermore, we didn't evaluate intellectual disability patients as a first step with Array CGH and other molecular-cytogenetic study. In addition, we did not perform functional studies to confirm the pathogenicity of VUS mutations. We didn't perform exome sequencing for their parents simultaneously. In addition, although genotype-phenotype correlation was confirmed, half of our patients didn't do sanger confirmation for patients and their parents. Most of our patients had severe disabilities and significant sequelae due to delayed diagnosis. At this stage of disease, starting treatments cannot reverse the previous damage inflicted on the developing brain. Furthermore, most of the patients did not have further follow-up visits to evaluate the treatment effect due to the global health crisis caused by COVID-19 pandemic (24). In addition, patients in developing countries face various other challenges such as the high cost of genetic testing and lack of insurance. We, therefore, have to recommend these tests to a more selected group of patients in such settings. It should also be noted that in many instances the families refuse further genetic testing due to the unavailability of an effective targeted therapy in most of the cases.

## Recommendation

Atypical cerebral palsy patients that require genetic studies including:

- Patients who had no risk factor for acquired cerebral palsy.
- Family history of same problems.
- Patients who have progressive symptoms and do not have any improvement in spite appropriate occupational therapy and physiotherapy.
- Patients who have normal brain MRI despite significant disability in various mental or motor areas.
- Patients with severe motor-mental disabilities.
- Patients with cerebral palsy who have severe and refractory seizures.
- Patients whose MRI shows abnormal lesions that are not usually seen on cerebral palsy.

## Data Availability Statement

The original contributions presented in the study are included in the article/supplementary material, further inquiries can be directed to the corresponding author/s.

## Ethics Statement

The studies involving human participants were reviewed and approved by Namazi Hospital, Shiraz University of Medical Science Ethics board. Written informed consent to participate in this study was provided by the participants' legal guardian/next of kin.

## Author Contributions

MF conceived and designed the study, collected, assembled, and interpreted NGS data. MN, SI, HN, and PK clinically evaluated the patients. MN drafted the manuscript. MF, AS, SB, FS, and ST performed the genetic studies. SI, HN, PK, AS, SB, FS, ST, and MF revised the manuscript. All authors contributed to the article and approved the submitted version.

## Conflict of Interest

The authors declare that the research was conducted in the absence of any commercial or financial relationships that could be construed as a potential conflict of interest.

## Publisher's Note

All claims expressed in this article are solely those of the authors and do not necessarily represent those of their affiliated organizations, or those of the publisher, the editors and the reviewers. Any product that may be evaluated in this article, or claim that may be made by its manufacturer, is not guaranteed or endorsed by the publisher.
